# Suicides among Danish cancer patients 1971–1999

**DOI:** 10.1038/sj.bjc.6602424

**Published:** 2005-03-01

**Authors:** U Yousaf, M-LM Christensen, G Engholm, H H Storm

**Affiliations:** 1 Department of Cancer Prevention and Documentation, Danish Cancer Society, Strandboulevarden 49, DK-2100 Copenhagen Ø, Denmark

**Keywords:** suicide, Danish cancer patients, cohort study, Danish cancer register, population based

## Abstract

Compared to the general population, the suicide risk among Danish cancer patients diagnosed in 1971–1986 was increased by 50% for men and 30% for women. We updated the earlier study to evaluate both long-term and recent trends in the suicide risk. Cancer patients with a first cancer diagnosed between 1971 and 1999 in Denmark were followed-up for completed suicide through 1999. Excluding nonmelanoma skin cancer, 564 508 cancer patients were included and 1241 suicides observed. Both the standardised mortality ratio (SMR) of suicide relative to the general population and the suicide rates were analysed with Poisson regression methods. The overall SMR was increased to 1.7 (95% CI. 1.6–1.9) for men and 1.4 (95% CI: 1.3–1.5) for women. Following the cancer diagnosis, the suicide risk was highest in the first 3 months for men and between months 3 and 12 for women. The risk was higher for nonlocalised cancer and for cancers with perceived poor prognosis. Breast cancer patients had a higher risk than other cancer patients with similar good prognosis. The suicide rates among cancer patients decreased with calendar time, but less so than the rates in the general population. The suicide risk among cancer patients has not decreased as much as in the Danish population and reasons for this should be explored. Breast cancer might be believed by patients to be more life threatening than it is. Assessment and treatment of depression could improve the quality of life for cancer patients who suffer from unrecognised depressions and in turn reduce the risk of suicide in cancer patients.

Somatic disease is a well-documented risk factor for completed suicide. Using register-based data, a 1.3–3 times increased suicide risk has been found among cancer patients in many countries (Louhivouri and Hakama 1979; Fox *et al*, 1982; Allebeck *et al*, 1989; Levi *et al*, 1991; Storm *et al*, 1992; Harris and Barraclough, 1994; Crocetti *et al*, 1998; Tanaka *et al*, 1999; Miccinesi *et al*, 2004). Studies on suicides in cancer patients have suggested that suicide risk is inversely correlated with time since diagnosis, increased with advanced cancer stage, higher for men than women, and varying with cancer site and age.

With 13 more years of incidence and follow-up, our study almost doubles the earlier study (Storm *et al*, 1992), which covered the risk of suicide and other violent deaths among 296 331 Danish cancer patients diagnosed in the period 1971–1986. Compared to the general Danish population, the observed 568 suicides represented increased suicide risks of 50% for men and 30% for women; risk was higher with nonlocalised cancers, and it increased by calendar time.

We conducted a detailed register and population-based cohort study among Danish cancer patients diagnosed and reported to the Danish Cancer Registry in the years 1971–1999.

## MATERIALS AND METHODS

All cancer patients identified in the Danish Cancer Register (Storm *et al*, 1997) with a first cancer, excluding nonmelanoma skin cancer, in the period 1971–1999, were followed up in the Danish Causes of Death Register (Juel and Helweg-Larsen, 1999). Follow-up was from the cancer diagnosis to the first of the following events: a second cancer, emigration, death, or end of study, 31 December 1999. We excluded patients with two simultaneously diagnosed cancers (3846 patients, 0.7%) or with a first cancer diagnosis reported on a death certificate alone or if the cancer was diagnosed at an autopsy (26 513 patients, 4.5%). Although primary outcome was suicide, we also examined other violent causes of death to evaluate a possible misclassification of suicides.

In addition to cancer site, gender, age and date of diagnosis, date of death, and cause of death, we also recorded the stage of the tumour at diagnosis as localised, regional spread, remote metastasis, or unknown. Cancer sites were analysed both grouped in broad ICD categories (Sundhedsstyrelsen, 2003), and according to the prognosis mesured by the 5-year relative survival as being high (>66%), medium (33–66%), and low (<33%) as shown in Table 1. (Storm and Engholm, 2002).

The person years at risk were grouped according to time since diagnosis into less than 3 months, 3–12 months, 1–2 years, 3–4 years, and 5 or more years, and stratified by age and calendar time in 5-year groups. The expected numbers of suicides were calculated by multiplying the risk time with the Danish population suicide rates in 5-year age and calendar time groups for each gender. The standardised mortality ratio (SMR) of suicide is defined as the observed divided by expected number of suicides, and the suicide rates as the observed number divided by the person years at risk.

The SMR and the rates were analysed using Poisson regression methods, assuming that the number of suicides follow a Poisson distribution. In the analysis of SMR, the logarithm of the expected number of suicides was used as the offset variable, while in the rate –analysis, the logarithm of the person years at risk was used.

In the analyses, the following factors were considered in the multiplicative models: age at cancer diagnosis, calendar time at diagnosis, the cancer site, time since diagnosis, and stage at diagnosis. For each factor, one of the categories was chosen as a reference, and the estimated parameters for the other categories interpreted as the relative risk compared to the reference, adjusted for the other factors in the model. The reference category was mostly chosen as the category with the highest number of suicides. Different types of categorisation of cancer sites were tried out: main ICD groups, individual cancer sites, and according to prognosis. Prognosis was divided into three groups using 5-year relative survival ratios for cancer patients in Denmark diagnosed during 1991–1995 (Storm and Engholm, 2002) as showed in Table 1. The 95% confidence intervals (95% CI) were reported, but a significance level of 1% was used due to the large data set and the relatively simple models. SAS version 8.02 was used for the analyses (SAS Institute INC, Cary, NC, USA).

## RESULTS

During the study period 1971–1999, a total of 564 508 cancer patients were included, contributing 861 713 person years at risk among men and 1 370 828 person years at risk among women.

### Suicide

A total of 1241 suicides (740 men, 501 women) were observed. The overall SMR of suicide was 1.7 (95% CI: 1.6–1.8) for men and 1.4 (95% CI: 1.3–1.5) for women.

#### Time since diagnosis

The adjusted relative risk (RR) of committing suicide was highest during the first 3 months after the cancer diagnosis for men and from 3 months to 1 year after the diagnosis for women, both when studying the SMR and the rates (Tables 2 and 3).

#### Calendar period at diagnosis

For the SMR, the RR was stable from 1971 to 1990, whereafter the RR increased until the end of the study period in 1999. The changes in SMR were significant for men but not for women. For rates, the adjusted RR decreased marginally during the study period (Tables 2 and 3).

#### Stage

For both genders, the adjusted RR of suicide increased by severity of clinical stage at the time of diagnosis, but not significantly for women. (Tables 2 and 3).

#### Cancer site

The adjusted RR of suicide according to cancer site was significantly elevated for men in the following main groups: Respiratory system, other specified sites, and Secondary and unspecified sites. For women, the RR was significantly elevated for Respiratory system, Breast, and Secondary and unspecified sites (results not shown).

We found a surprisingly good fit of the models with cancer sites according to prognosis divided into good, medium, and low relative survival. The RR of suicide was highest among patients with poor prognosis cancer, and also when we adjusted for the other risk factors (Tables 2 and 3). Breast cancer had a deviating pattern with a higher suicide risk than other cancer sites with good prognosis (Table 3).

#### Age at diagnosis

The adjusted RR increased with age for men and was highest for women at ages 50–79 years in the rate models, while no significant variation was found in the SMR models (Tables 2 and 3).

#### Interactions

We checked for interactions between risk factors, but found no significant interactions at the 1% level.

### Other violent deaths

In total, 3038 (1349 for men and 1689 for women) violent deaths other than suicide were observed. The SMR of death by traffic accident and by accidental poisoning was not significantly elevated. The SMR of death due to murder or other violence, and to other accidents than fall accidents was significantly elevated, but since the numbers of cancer patients with these outcomes were small (217 among men and 192 among women), no further analyses were made (data not shown).

#### Death by fall

Death by fall accidents constituted the majority of other violent deaths, 878 among men and 1329 among women. The overall SMR of death by fall accident was 1.2 (95% CI: 1.2–1.3) for men and 1.2 (95% CI: 1.2–1.3) for women. The RR was highest right after the cancer diagnosis, and more so among patients with metastatic cancer. The risk increased during the study period; it was highest at ages 50–64 years, and among patients with poor prognosis cancer. All factors were significant in the adjusted models among women and, except for period at diagnosis, also for men.

## DISCUSSION

The overall suicide risk of cancer patients relative to the population risk in our study was marginally elevated compared to the RR reported by Storm *et al* (1992).

### Time since cancer diagnosis

The strongest risk factor for suicide was time since the cancer diagnosis. The increased suicide risk following diagnosis shows the need for psychosocial care and support. Many cancer patients are going through a crisis trying to cope with the fact that they have a life-threatening disease. Tanaka *et al* (1999) found the highest suicide risk between the third and fifth month following diagnosis and the suicides committed in this period were immediately after discharge from hospital. Difficulties of psychological adjustment without the support of the hospital environment might be one possible explanation. We similarly discovered a peak in suicides between 3 and 12 months after diagnosis for women, whereas the highest suicide risk for men was within the first 3 months following diagnosis. Time since cancer diagnoses was only borderline significant among women after adjusting for the other risk factors. The different suicide pattern seen for gender may be explained by a higher success rate of suicide attempts among men as they, in general, use more determined suicide methods than women (Sundhedsstyrelsen, 1998).

The suicide risk remained elevated more than 5 years after diagnosis, which could be explained by terminally ill patients who commit suicide to avoid suffering in the last phases of their illness, or by recurrent or metastatic disease developing. These states are not recorded by the cancer registry and it is thus not possible to study using register data.

### Study period

The suicide rates in the general Danish population have changed dramatically during our study period. The suicide rate peaked in Denmark in 1981–1985 with a world age standardized rate per 100 000 of 30 for men and 16 for women. It has been falling steadily ever since – to 17 for men and 6 for women in 1996–1999 (Table 4).

The suicide rates for cancer patients also decreased, but not as much as seen in the general population; hence, the SMR for suicide increased for cancer patients during our study period. This trend was also observed by Storm *et al* (1992) for the period up to 1986.

Although suicide might seem rational for the terminally ill patient or for patients with poor prognosis, a suicide is rare in the absence of a psychiatric disorder, even among cancer patients (Hietanen and Lönnquist, 1991; Filiberti *et al*, 2001).

The prevalence of depression in the cancer population has been estimated to be 10–30% depending on the diagnostic criteria used (Chochinov *et al*, 1994; Breitbart *et al*, 2000; Ciaramella and Poli, 2001).

The increased suicide risk points to the importance of health professionals recognising the symptoms of depression in cancer patients and acting accordingly. Diagnosis of depression in cancer patients can be challenging because sadness is part of a natural emotional reaction of being diagnosed with cancer, and many nonspecific somatic symptoms frequently associated with depression can result from the cancer itself. The increasing SMR for suicide among cancer patients during our study period could indicate that depression among Danish cancer patients is underdiagnosed and undertreated as seen by Tiernan *et al* (2002).

The increasing SMR for suicide with calendar time in Danish cancer patients is in contrast with a recently published Italian study based on 102 cancer suicides. The SMR for suicide decreased with calendar time from 1985 to 1999 in Central Italy, which is a low incidence suicide area. Miccinesi *et al* (2004) proposed that better treatment options and better communication of diagnosis were possible explanations of their finding. The SMR calculated in the Miccinesi study was only adjusted for time since diagnosis, and only analysed for men and women combined.

The first cancer counselling centre in Denmark, offering information and psychological counselling to patients and relatives, was founded in 1978 in Copenhagen. Today every county in Denmark has a cancer counselling centre and the major oncology departments have a psychologist who educates the staff in dealing with psychosocial problems. Furthermore, a 5-day course in communication is now mandatory in the postgraduate training of doctors, so the psychosocial aspect of a cancer diagnosis has become much more recognised during our study period. All of this should ideally lead to a lower suicide risk than before in cancer patients, approximating that of the general population; however, this was not observed. One may speculate whether the increased openness and the demand on patients to be involved in the decision process about their therapy lead to unwarranted outcomes such as suicides.

### Stage, prognosis, and cancer site

The suicide risk increased with the severity of clinical stage at the time of diagnosis as seen by others (Louhivouri and Hakama, 1979; Storm *et al*, 1992; Tanaka *et al*, 1999), for news of metastatic cancer must be a shock. Furthermore, widespread cancer may cause profound pain and physical impairment, besides a poor prognosis. Different sites have been associated with the highest suicide risks, including gastrointestinal cancer (Louhivouri and Hakama, 1979), respiratory and gastrointestinal cancers (Allebeck *et al*, 1989), genital cancers (Tanaka *et al*, 1999), and stomach, rectum, kidney, brain and nervous system cancers (Storm *et al*, 1992). The differences probably reflect that the numbers associated with each site are small even in large cohorts; therefore, the risk estimates are unstable.

Cancers with a poor prognosis, here measured by low 5-year relative survival ratio, yielded the highest risks of suicide. Aggregating cancer diagnosis in three groups according to prognosis and using this factor together with stage in the analyses showed a good fit of the models. Female breast cancer patients had a good prognosis, but a high suicide risk, even when adjusted for stage. For women we improved the model fit by keeping breast cancer as a separate category in the prognosis factor and found no interaction with stage. The influence of surgery, especially mastectomy, on psychosocial and psychosexual perception may play a role in suicide by breast cancer patients. An American study of breast cancer patients showed major depressive disorder among 9% and distress among 29% (Coyne *et al*, 2004) and suggested that diagnosis and treatment of breast cancer have a big influence on psychosocial functioning. Breast cancer patients may consider their disease more life-threatening than it is.

We excluded nonmelanoma skin cancer because it is not considered a life-threatening disease. Whether such patients do have a similar suicide risk to the population risk will be evaluated in another paper.

### Age at diagnosis

Older age and depressed mood are important risk factors for suicide in the population, with highest risk for men above 80 years of age and for women aged 50–79 years (Akechi *et al*, 2000, Erlangsen, 2004), perhaps because suicide attempts are often more successful for men. A similar pattern was found among cancer patients. Age was significant in modelling of the rates as seen in Tables 2 and 3, but insignificant in models where the risk in the cancer population was compared to the population risk, that is, the SMR models.

### Validity of suicide statistics

We investigated whether the high relative risk of dying from falls, especially for women, could be hidden suicides. Our findings support this idea. The high SMR for death from falls immediately after cancer diagnosis and increasing with severity of clinical stage follows the same pattern as the cancer patients’ SMR for death by suicide. The relative risk of death from falls was highest at ages 0–49 and 50–64 years for both men and women. If the excess risk of such deaths was ascribed to hidden suicides, this would maximally increase the total number of suicides by 8% for men and 14% for women. Other plausible explanations exist such as side effects of cancer medications, delirious states, and general weakness.

The Danish suicide rates have always been high compared with most other countries. In Denmark, suicide is not a criminal act. Furthermore, as a secular society, suicide is not connected with strong religious condemnation. All cases of unexpected and violent deaths are examined by the police and a specialist in forensic medicine (Atkinson *et al*, 1975; Hessø, 1987; Kolmos and Bach, 1987). Kolmos and Bach (1987) searched the Danish registers for possible misclassifications related to poisoning and drowning, and concluded that the suicide statistics in Denmark are reliable and come close to reflecting the true suicide rate. Misclassification of suicide as accidental falls has only been suggested by Storm *et al* (1992) and this study.

## CONCLUSION

Our study included the 1241 suicides in a cohort of 564 517 Danish cancer patients diagnosed between 1971 and 1999, which makes it the largest study to date on suicide and cancer. It is based on the Danish Cancer Register (Storm *et al*, 1997), one of the most complete nationwide cancer registers in the world, and the Danish Register of Causes of Death (Juel and Helweg-Larsen, 1999). Our study is a cohort study based on linkage of independent registers, thus avoiding information bias and recall bias.

We have shown that a diagnosis of cancer is a risk factor of suicide for both genders, and we identified certain groups among cancer patients at very high risk of committing suicide: the suicide risk was higher for men than for women. The suicide risk was highest the year following diagnosis, but remained elevated even after 5 years from diagnosis. Cancer patients diagnosed between 50 and 79 years of age committed the majority of suicides. A diagnosis of cancer with poor prognosis and cancer with metastases at the time of diagnosis gave a high relative risk of suicide. The SMR of suicide increased with calendar time, although the total number of suicides decreased. Our results are in agreement with other studies on the link between cancer and suicide and suicide and physical disease in general (Louhivouri and Hakama, 1979; Fox *et al*, 1982; Allebeck *et al*, 1989; Levi *et al*, 1991; Storm *et al*, 1992; Harris and Barraclough, 1994; Stenager *et al*, 1994; Crocetti *et al*, 1998; Stenager *et al*, 1998; Tanaka *et al*, 1999; Miccinesi *et al*, 2004). The marginal and adjusted analyses of SMR of suicide and the analyses of the rates among cancer patients show the stability of the results and facilitate comparison with most other studies.

The total number of suicides among cancer patients was not large, an average of 43 suicides per year with an excess number of 10–15, but it may be seen as an indicator of a high frequency of shock and depression among cancer patients. We had no possibility of controlling for other known risk factors of suicide, such as unemployment, psychiatric disease, marital status, drug- and/or alcohol abuse, and former suicide attempts (Mortensen *et al*, 2000), as these factors are not routinely collected by the registers used. Further research in this area should include a linkage study between the Danish Cancer Register (Storm *et al*, 1997), the Danish Psychiatric Register (Munk-Jørgensen and Mortensen, 1997), the Danish National Hospital Register (Andersen *et al*, 1999), and the Register of Causes of Death (Juel and Helweg-Larsen, 1999), in order to compare the prevalence of psychiatric disorders in cancer patients committing suicide relative to other cancer patients. Nested case control studies could assess a possible connection between suicide more than a year after cancer diagnosis and recurrent or metastasised cancer or treatment failure, and the association between discharge from hospital and increased suicide risk during the first year after a cancer diagnosis should be studied.

In clinical practice, it is important to be aware that depression and suicidal ideation in cancer patients are common and that cancer patients are at an increased risk of suicide. Assessment and treatment of depression could improve the quality of life for cancer patients who suffer from unrecognised depressions and, in turn, reduce the risk of suicide in cancer patients.

## Figures and Tables

**Table 1:** Cancer sites grouped according to prognosis by cancer site measured by good (>66%), medium (33–66%), and poor (<33%) 5-year relative survival in Denmark for cancers diagnosed during 1991–1995

**Prognosis 5-year relative survival**	**Cancer sites**
Good (>66%)	Lip, breast, cervix, corpus, testis, melanoma skin, thyroid, Hodgkin's disease
	
Medium (33–66%)	Salivary glands, mouth, colon, rectum, nasal cavity, larynx, uterus, prostate, kidney, bladder, eye, brain and nervous system, endocrine glands, bone, connective tissue, non-Hodgkin's lymphoma, leukaemia, mycosis fungoides
	
Poor (<33%)	Tongue, pharynx, oesophagus, stomach, small intestine, liver, gall bladder, pancreas, peritoneum, lung, pleura, ovary, metastases, multiple myeloma

**Table 2:** Suicides among men with a first cancer, excluding nonmelanoma skin cancer, in the Danish Cancer Register 1971–1999

				**Models of SMR**		**Model of rates**
	**Number observed (*N*=740)**	**Number expected (*N*=439.7)**	**Person-years (%) (*N*=861713)**	**Univariate RR (95% CI)**	**Multivariate RR (95% CI)**	**Multivariate RR (95% CI)**
*Intercept*					1.2 (1.0–1.5)	(7.4 (5.9–9.2))^*^10^−4^
*Time since diagnosis*						
Intercept/*P*-value				1.2 (1.0–1.4)/*P*<0.00001	*P*<0.00001	*P*<0.00001
<0.25 year	117	30.2	6.7	3.3 (2.6–4.1)	2.4 (1.9–3.1)	2.4 (1.9–3.1)
0.25–<1 year	140	63.1	14.1	1.9 (1.5–2.3)	1.5 (1.2–1.9)	1.5 (1.2–1.9)
1–<3 year	185	106.2	24.0	1.5 (1.2–1.8)	1.3 (1.1–1.6)	1.3 (1.1–1.6)
3–<5 year	95	69.0	15.6	1.2 (0.9–1.5)	1.1 (0.8–1.4)	1.1 (0.8–1.4)
5+ year	203	171.2	39.6	1	1	1
						
*Stage of cancer*						
Intercept/*P*-value				1.4 (1.3–1.5)/*P*<0.00001	*P*=0.002	*P*=0.005
Localised	408	294.5	66.3	1	1	1
Regional	102	51.8	12.0	1.4 (1.1–1.8)	1.1 (0.9–1.3)	1.1 (0.8–1.3)
Metastasised	78	27.6	6.2	2.0 (1.6–2.6)	1.4 (1.1–1.8)	1.4 (1.1–1.8)
Unknown	152	65.9	15.5	1.7 (1.4–2.0)	1.4 (1.2–1.7)	1.4 (1.1–1.7)
						
*Period of diagnosis*						
Intercept/*P*-value				1.7 (1.4–1.9)/*P*<0.00001	*P*=0.004	*P*=0.09
1971–1975	93	82.6	17.5	0.7 (0.5–0.9)	0.7 (0.5–0.9)	0.7 (0.5–0.9)
1976–1980	162	97.9	20.7	1	1	1
1981–1985	158	96.6	20.6	1.0 (0.8–1.2)	0.9 (0.8–1.2)	0.9 (0.8–1.2)
1986–1990	150	85.3	19.9	1.1 (0.9–1.3)	1.0 (0.8–1.2)	0.9 (0.7–1.1)
1991–1995	121	58.5	15.6	1.3 (1.0–1.6)	1.1 (0.9–1.4)	0.8 (0.7–1.1)
1996–1999	56	18.8	5.7	1.8 (1.3–2.4)	1.3 (0.9–1.8)	0.8 (0.6–1.1)
						
*Age at diagnosis (years)*						
Intercept/*P*-value				1.7 (1.5–1.9)/*P*=0.17	*P*=0.07	*P*=0.005
0–49	115	81.2	25.9	0.8 (0.7–1.0)	1.2 (0.9–1.5)	0.7 (0.6–0.9)
50–64	237	130.3	30.9	1.1 (0.9–1.3)	1.2 (1.0–1.4)	1.0 (0.8–1.2)
65–79	314	184.1	37.0	1	1	1
80+	74	44.2	6.3	1.0 (0.8–1.3)	0.8 (0.7–1.1)	1.3 (1.0–1.6)
						
*Prognosis of cancer site*						
Intercept/*P*-value				1.6 (1.5–1.7)/*P*<0.00001	*P*<0.00001	*P*<0 .00001
Good (+66%)	70	73.7	19.7	0.6 (0.5–0.8)	0.6 (0.5–0.8)	0.6 (0.5–0.8)
Medium (33–66%)	183	60.4	66.9	1	1	1
Poor (0–33%)	487	305.7	13.4	1.9 (1.6–2.3)	1.6 (1.3–1.9)	1.6 (1.3–1.9)

Multivariate models included the factors time since diagnosis, stage of cancer, period of diagnosis, age at diagnosis, and prognosis of cancer site measured by 5-year relative survival.

Models of standardised mortality ratio (SMR) and rates showing relative risk (RR) in both univariate and multivariate models.

**Table 3:** Suicides among women with a first cancer, excluding nonmelanoma skin cancer, in the Danish Cancer Register 1971–1999

				**Models of SMR**		**Model of rates**
	**Number observed (*N*=501)**	**Number expected (*N*=361.4)**	**Person-years (%) (*N*=1370828)**	**Univariate RR (95% CI)**	**Multivariate RR (95% CI)**	**Multivariate RR (95% CI)**
*Intercept*					1.0 (0.7–1.3)	(2.8 (2.1–3.8)) ^*^10^−4^
*Time since diagnosis*						
Intercept/*P*-value				1.1 (1.0–1.3)/*P*=0.00003	*P*=0.02	*P*<0.00001
<0.25 year	32	17.6	4.6	1.6 (1.1–2.4)	1.3 (0.9–1.9)	1.7 (1.1–2.5)
0.25–<1 year	86	41.2	10.9	1.9 (1.5–2.4)	1.6 (1.2–2.1)	2.0 (1.6–2.7)
1–<3 year	127	79.3	21.2	1.4 (1.2–1.8)	1.3 (1.0–1.6)	1.6 (1.3–2.0)
3–<5 year	71	57.2	15.5	1.1 (0.9–1.5)	1.0 (0.8–1.4)	1.2 (0.9–1.6)
5+ year	185	166.1	47.8	1	1	1
						
*Stage of cancer*						
Intercept/*P*-value				1.3 (1.1–1.4)/*P*<0.00001	*P*=0.26	*P*=0.30
Localised	318	253.9	69.6	1	1	1
Regional	97	63.1	17.6	1.2 (1.0–1.5)	1.0 (0.8–1.3)	1.0 (0.8–1.3)
Metastasised	29	12.0	3.2	1.9 (1.3–2.8)	1.4 (0.9–2.1)	1.4 (0.9–2.1)
Unknown	57	32.4	9.6	1.4 (1.1–1.9)	1.3 (0.9–1.7)	1.2 (0.9–1.6)
						
*Period of diagnosis*						
Intercept/*P*-value				1.3 (1.0–1.5)/*P*=0.0009	*P*=0.10	*P*=0.08
1971–1975	94	83.7	19.8	0.9 (0.7–1.2)	0.9 (0.7–1.2)	1.0 (0.7–1.3)
1976–1980	112	89.3	21.7	1	1	1
1981–1985	118	80.7	21.0	1.2 (0.9–1.5)	1.1 (0.9–1.5)	1.1 (0.8–1.4)
1986–1990	82	62.0	18.9	1.1 (0.8–1.4)	1.0 (0.7–1.3)	0.8 (0.6–1.0)
1991–1995	73	36.7	14.1	1.6 (1.2–2.1)	1.4 (1.0–1.9)	0.8 (0.6–1.1)
1996–1999	22	9.0	4.6	2.0 (1.2–3.1)	1.5 (0.9–2.4)	0.6 (0.4–1.0)
						
*Age at diagnosis*						
Intercept/*P*-value				1.5 (1.3–1.7)/*P*=0.70	*P*=0.80	*P*=0.04
0–49	120	92.0	30.2	0.9 (0.7–1.1)	1.0 (0.8–1.3)	0.8 (0.6–1.0)
50–64	193	142.4	35.4	0.9 (0.7–1.1)	1.0 (0.8–1.2)	1.0 (0.8–1.2)
65–79	164	109.8	28.8	1	1	1
80+	24	17.2	5.6	1.0 (0.6–1.4)	0.8 (0.5–1.3)	0.7 (0.4–1.1)
						
*Prognosis of cancer site*						
Intercept/*P*-value				1.5 (1.3–1.7)/*P*=0.0001	*P*=0.002	*P*=0 .0005
Good (+ 66%)	104	97.7	27.3	0.8 (0.6–1.1)	0.9 (0.7–1.2)	0.9 (0.7–1.2)
Medium (33–66%)	120	94.7	27.0	1	1	1
Poor (0–33%)	78	34.7	9.4	1.8 (1.3–2.4)	1.6 (1.2–2.1)	1.7 (1.2–2.2)
Breast cancer	199	134.2	36.4	1.2 (0.9–1.5)	1.2 (1.0–1.5)	1.3 (1.0–1.6)

Multivariate models included the factors time since diagnosis, stage of cancer, period of diagnosis, age at diagnosis and prognosis of cancer site measured by 5-year relative survival.

Models of standardised mortality ratio (SMR) and rates showing relative risk (RR) in both univariate and multivariate models.

**Table 4:** Number of suicides in Denmark per year and age-standardised (world) suicide rates (WSTP) per 100 000 in 1971–1999

	**Men**		**Women**	
**Calendar year**	**Observed number per year**	**Rate (WSTP)**	**Observed number per year**	**Rate (WSTP)**
1971–1975	766	26.2	460	14.8
1976–1980	817	27.4	498	15.5
1981–1985	931	30.2	542	16.3
1986–1990	868	26.7	493	14.0
1991–1996	711	21.1	364	9.4
1996–1999	575	16.8	233	6.1
